# Catastrophic health expenditure: a comparative study between hypertensive patients with and without complication in rural Shandong, China

**DOI:** 10.1186/s12889-020-08662-0

**Published:** 2020-04-22

**Authors:** Xinyi Zhang, Qiongqiong Xu, Xiaolei Guo, Zhengyue Jing, Long Sun, Jiajia Li, Chengchao Zhou

**Affiliations:** 1 School of Public Health, Shandong University, Jinan, 250012 China; 2 Shandong Center for Disease Control and Prevention, Jinan, 250014 China; 3 NHC Key Lab of Health Econonics and Policy Research, Shandong University, Jinan, 250012 China

**Keywords:** Catastrophic health expenditure, Hypertension, Complication, Determinants, China

## Abstract

**Background:**

Some previous studies have assessed catastrophic health expenditure (CHE) in households with hypertensive patients, but few have examined the difference of CHE in hypertensive patients with and without complications. The purpose of this study is to compare the incidence and determinants of CHE between hypertensive patients with and without complications.

**Methods:**

Data of this study were from a cross-sectional study in Shandong Province in China in 2016. Of the recruited 3457 hypertensive patients registered in the NCDs management system in the sampling villages, 3113 completed the survey, with a response rate of 90.05%.CHE was defined as out-of-pocket payments for hypertensive care that equaled or exceeded 40% of the household capacity to pay (non-food expenditure). Hypertension complications (e.g., stroke, coronary heart disease, hypertensive kidney disease, etc.) were collected in this study, which was categorized into 0 (no), 1(single), and 2 and more according to the types of hypertensive complications. We employed Chi-square test to explore associated factors and logistic regression model to identify the determinants of CHE.

**Results:**

The incidence of CHE and impoverishment is 13.6 and 10.8% among hypertensive patients. The incidence of CHE with one complication is 25.3% (*Ρ* = 0.000, OR = 2.29) and 47.3% (*P* = 0.000, OR = 3.60) in patients with two or more complications, which are both statistically higher than that in patients without complication (6.1%). Across all types of patients, income levels are inversely related to the incidence of CHE. Patients who use outpatient or inpatient service are more likely to experience CHE (*Ρ* = 0.000). Factors including living arrangements, family size, educational attainment are found to be significantly associated with CHE in some subgroups (*Ρ* <0.05).

**Conclusions:**

CHE and impoverishment incidence among hypertensive patients are both high in rural China. Patients with hypertensive complication are at higher catastrophic risk than those without complication. More attention needs to be paid to households with hypertension patients, especially for those with hypertension complications.

## Background

Cardiovascular disease is the world’s leading cause of deaths, accounting for one third of the total [1]. Hypertension is one of the major risk factors for cardiovascular disease, which plays a major role in the development of cerebrovascular disease, cardiac and renal failure [2]. About 50% of coronary heart disease and 75% of the burden of cerebrovascular disease are caused by hypertension [3]. Globally, approximately one billion people suffer from hypertension. It is estimated that the incidence of hypertension will rise from 26.4 to 29.2% in the world by 2025 [2, 4]. Hypertension and its complications are the leading causes of death and disability [5] . The number of deaths worldwide due to hypertensive complication is 9.4 million, of which 45% die from heart disease, and 51% die from stroke [6]. The World Health Organization (WHO) ranks hypertension as one of the top five health risk factors [5].

In China, the prevalence of hypertension in adults aged 18 years and older was 27.9%, with more than 250 million patients, and the number of new cases per year about 10 million. This prevalence was even higher (32.5%) among the adults aged 35 to 74 [7–9]. It was estimated that 2.5 million Chinese adults died from hypertension and its complications, accounting for 28% of all deaths in 2013 [10]. China’s total health expenditure was US$ 30.57 billion in 2013, of which the direct economic burden of hypertension accounted for 6.61% [11] . Chinese Health Statistics estimated that the out of pocket (OOP) expenditure of residents was US$ 16.39 billion in 2014, which means OOP that payments remain relatively high in China [12]. Previous studies also showed that households with a large proportion of OOP expenses were extremely vulnerable to financial difficulties, especially those with chronic diseases [13]. In the past decades, the Chinese government has introduced several social health insurance schemes (i.e., Basic Medical Insurance Scheme for Urban Employees, Medical Insurance for urban and rural residents) to the urban and rural residents, and also medical financial assistance to the poor, so as to reduce economic burden of the households with patients [14] . However, the households which experiencing hypertension and hypertension complications (i.e., heart attack, stroke and kidney failure) often spend a substantial share of their income on hypertension-related healthcare use [4] . Previous study in China showed that hypertension and its complications ranked the first of all diseases with heavy economic burden among the rural residents [15] . In addition, rural residents are found to be of higher economic risk than urban residents [16, 17] . For rural households in China, the emergence of such diseases might lead to severe economic risks and a further induced poverty [18]. The fundamental role of a healthcare system is not only to facilitate access to health services when needed, but also protect households from financial catastrophe associated with illness [19] .

However, many people still do not have access to the healthcare services they needed owing to economic conditions [20]. When healthcare expenditure seriously affects household living conditions, or even push the household into poverty, we call it “catastrophic” [21], which can be quantified by catastrophic health expenditure (CHE). Generally, we define CHE as out-of-pocket payment (OOP) for health care that exceed a specified proportion of household income, with the consequence that the household may sacrifice the consumption of other goods or services necessary for their well-being [21, 22]. In China, several studies have explored the CHE among the general populations. A study using a national representative data by Li et al. found that the incidence of CHE among the general population in China was 13.0%, which was higher than that in some other low-income countries [23]. Households with members suffering from chronic diseases have a greater possibility of experiencing catastrophic health expenditure than their counterparts, which is consistent in both developed countries and developing countries [23, 24]. Under a rapid increasing trend in prevalence of hypertension, to identify the incidence and determinants of CHE for hypertension patients in rural areas, and evaluate the effect of hypertension complications on the incidence, intensity of CHE are of high priority for protecting households with hypertension from financial catastrophe in rural China.

To date, there are only very few studies have explored the CHE in hypertensive patients, and no studies have assessed the effect of complications on the incidence, intensity of CHE in China. The overall objective of this study is to compare CHE between hypertensive patients with and without complications. To do so, we have several specific objectives. Firstly, we would compare CHE between hypertensive patients with complications and without complications. Secondly, we will identify the determinants for CHE in the hypertensive patients in rural China. Like China, growing incidence of hypertension and co-payment for its health expenditure are also issues in other low and middle income countries (LMICs), the findings from this study is also of high significance to other LMICs.

## Methods

### Study participants

Data for the current analysis came from a cross-sectional survey our research team conducted in Shandong Province, which was the second largest province in China. The prevalence of hypertension in Shandong residents aged 18–69 in 2013 was 27.9%, significantly higher than the national average level [25]. An expected incidence of 10% of CHE and also a tolerance error of 0.15 were used to estimate the sample size, and at least 1600 hypertensive patients should be investigated. A 3-stage cluster sampling was employed to select participants in this survey. Firstly, according to Gross Domestic Product (GDP) per capita in Shandong in 2015, we stratified all counties into three groups. We selected four counties as the study sites, one (Rushan) from the upper level, two (Yiyuan and Gaotang) from the medium level, and one (Liangshan) from the lower level. Secondly, likewise, we stratified all townships into three groups in each selected county, from each group we chose one township. Thirdly, we then randomly chose two villages with a size of permanent residents over 1000 from each selected township. More details about data collection methods were described in our previous studies [26].

According to the Chinese guideline for the management of hypertension, the criterion for hypertension was systolic blood pressure (SBP) ≥ 140 mm HG (1 mm HG = 0.133 k Pa) and diastolic blood pressure (DBP) ≥ 90 mm HG [27]. In this study, the status of hypertension and also its complications were diagnosed by medical professionals and registered in the sample village chronic case management system. We have collected the hypertension status and its complications of the participants from the chronic case management system in the sampling villages. In order to validate the information, we asked the respondents about whether they were diagnosed with hypertension complications and further for specific complications again when conducting face-to-face interview. All of the 3457 hypertensive patients under NCDs case management in the sampling communities or villages were recruited in this survey. In total, 3113 rural hypertensive patients complete the interview and were included in current study, with a response rate of 90.05%.

### Data collection

All the subjects were interviewed face-to-face using a standard structured questionnaire by trained postgraduate students from Shandong University School of Public Health. The questionnaire used in this study has been published elsewhere [28]. In order to ensure a high response rate, about 1 week before the normal survey, the sampling hypertensive patients were informed of the purpose and time of the interview by the local healthcare practitioners, and signed the willingness for the participation in the survey. To achieve the purposes of this study, completed questionnaires were subject to stringent quality assurance in each day. The questionnaire included the patients’ basic information (gender, age, marital status, education attainment, household composition), household income, total household expenditure, food expenditure, OOP payments for hypertension-related healthcare, health insurance status, and out-patient service and in-patient service. When conducting the interview, the main information of the interviewed hypertensive patients in the case management system would be also reviewed by the interviewers to validate the response of the interviewees.

### Variables and definitions

#### Outcome and measurement

In our study, we define CHE as OOP (out-of-pocket healthcare payments) that equal or exceed 40% of the household capacity to pay [29], of which, the household capacity to pay means that household total expenditure minus food expenditure [30, 31] . OOP expense for hypertension and its complications refers to the direct expenses paid to the medical service provider of the whole household, excluding indirect costs such as loss of working time, transportation and accommodation fees incurred at the time of medical treatment [32, 33]. We usually use incidence and intensity indicators to assess CHE [34, 35]. The incidence of CHE can be expressed by head count (HC) and intensity indicators can be evaluated by mean gap (MG) and mean positive gap (MPG). HC is estimated as follows:

$$ HC=\frac{1}{N}\sum \limits_{i=1}^NE $$

Where N is the sample size, E is an indicator equal to 1 if OOP of a household i as a proportion of its capacity to pay is greater than the threshold Z and zero otherwise. MG is the average amount by which payments, as a proportion of income, equals or exceeds 40% of their capacity to pay. It is estimated as follows:

$$ MG=\frac{1}{N}\sum \limits_{i=1}^NG $$

Where N is the sample size, G is an indication of how much OOP exceed the threshold, equal to Ti / Xi-Z if Ti / X > Z and zero otherwise. Here, Ti is the OOP payments of household i, Xi is the household capacity to pay and Z is the threshold share. MPG is defined as the head count being a fraction of the MG. In addition, the impoverishment effect due to hypertension-related OOP payment is also assessed in this study, which is measured by the difference between the relevant pre-payment and post-payment measures. The specific calculating methods of CHE incidence and intensity, impoverishment incidence are described in detail by Wagstaff and colleagues elsewhere [22].

### Independent vairables and covariates

#### Hypertensive complication

A question of “Have you ever been diagnosed with hypertension complication (including stroke, coronary heart disease, hypertensive kidney disease, heart disease etc.)?” was used to measure hypertension complication in this study. If the answer was ‘yes’, we will record the specific disease(s) in details. We categorized the types of hypertensive complications into 0 (no), 1(single), and 2(multiple complications).

### Hypertension-related variables

Hypertension-related variables included duration of hypertension (≦5, 5-, 10-, > 15 years), hospitalization due to hypertension and its complication in the past year (yes vs. no), and out-patient service (yes vs. no).

### Social demographic characteristics

The social demographic characteristics of the participants included age, gender, marital status, educational attainment, health insurance status, head of household, gender of household head, family size, and household living arrangements.

### Economic status

Economic status included household income (Q1, Q2, Q3, Q4, and Q1 was the poorest, and Q4 was the richest), Dibao (yes vs. no), debt (yes vs. no). We used a question of “How much was the total income of your household in the past year (urban households were disposable income, rural households were net income)?” to measure the yearly household income.

### Data analysis

The data is double entered and checked using EPI Data 3.1. The statistical package SPSS 22.0 is used to analyze the data. Household total expenditure, food expenditure, capacity to pay and OOP payments are presented as means and medians. Logistic regression analysis is used to compare the incidence of CHE between hypertensive patients with and without complications, Chi-square test is used to explore which factors are associated with CHE. Multivariate logistic regression analysis is employed to assess the determinants for CHE in each type of subgroups. Statistical significance is set at the 5% level. Sampling weights were included in all analyses to deal with potential cluster effects.

## Results

### Demographic characteristics

We present the descriptive statistics of the study participants in Table 1. Of the 3113 hypertensive patients, 2009(64.5%) have no complication, 1013(32.5%) have one complication, and 91(3%) have two or more complications. Majority of the study participants are women (63.3%), with an education level of elementary school and below (78.7%). As for the living arrangements, empty-nest couples account for 65.2%, while empty-nest single account for 14.1%. During the 12 months prior to survey, 414(13.3%) patients used in-patient service, 410(13.2) patients used out-patient service. CHE was different across different types of economic status (*Ρ* < 0.001), number of complications (*Ρ* < 0.001), education level (*Ρ* < 0.05), duration of disease (*Ρ* < 0.01), in-patient service (*Ρ* < 0.001), outpatient service (*Ρ* < 0.001), living arrangement (*Ρ* < 0.05).

**Table 1:** Socio-demographic characteristics of study participants in rural Shandong, China, 2016

Characteristics	N (%)	OR	95%CI
**Observations**	3113 (100)	–	–
**Age (years)**			
≤ 45	60 (1.90)	1.0	
45–60	787 (25.3)	3.01	0.72–12.57
60–70	1338 (43.0)	4.42	1.07–18.25^*^
> 70	928 (29.8)	6.46	1.56–26.70^*^
**Gender**			
Male	1142 (36.7)	1.0	
Female	1971 (63.3)	1.17	0.94–1.45
**Marital status**			
Single	64 (2.1)	1.0	
Married	2571 (82.6)	1.98	0.72–5.49
Bereft of spouse ^a^	478 (15.4)	4.92	1.75–13.82^**^
**Complications**			
0	2009 (64.5)	1.0	
1	1013 (32.5)	5.18	4.12–6.53^***^
≥ 2	91 (3.0)	13.736	8.76–21.55^***^
**Education**			
None	1391 (44.7)	1.0	
Primary school	1057 (34.0)	0.61	0.48–0.76^***^
Junior school	517 (16.6)	0.49	0.35–0.68^***^
Senior school or above	148 (4.8)	0.58	0.34–0.99^*^
**Household income**			
Q4^b^	755 (24.2)	1.0	
Q1	783 (25.2)	3.75	2.69–5.21^***^
Q2	774 (24.9)	2.17	1.53–3.07^***^
Q3	801 (25.7)	1.78	1.24–2.53^*^
**Health insurance**^**c**^			
URBMI	2983 (95.8)	1.0	
UEBMI	40 (1.3)	0.33	0.08–1.36
CMI	11 (0.4)	0.62	0.08–4.87
Others ^d^	79 (2.5)	0.51	0.22–1.18
**Duration of disease (years)**			
≤ 5	1031 (33.1)	1.0	
5–10	1050 (33.7)	1.26	0.96–1.65
10–15	397 (12.8)	1.74	1.25–2.41^**^
> 15	635 (20.4)	1.83	1.38–2.43^***^
**In-Patient**			
No	2699 (86.7)	1.0	
Yes	414 (13.3)	16.58	12.99–21.14^***^
**Out-Patient**			
No	2703 (86.8)	1.0	
Yes	410 (13.2)	3.29	2.58–4.20^***^
**Dibao**^e^			
No	2943 (94.5)	1.0	
Yes	170 (5.5)	2.29	1.59–3.29^***^
**Be in debt**			
No	2707 (87.0)	1.0	
Yes	406 (13.0)	0.99	0.66–1.24
**Head of household**			
No	1424 (45.7)	1.0	
Yes	1689 (54.3)	1.31	1.06–1.61^*^
**Head of household’s gender**			
Male	2415 (77.6)	1.0	
Female	698 (22.4)	1.71	1.36–2.14^*^
**Family size**			
≤ 4	2858 (91.8)	1.0	
> 4	255 (8.2)	0.41	0.24–0.68^**^
**Living arrangement**			
Others	642 (20.6)	1.0	
Empty-nest single	440 (14.1)	4.32	2.99–6.24^***^
Empty-nest couple	2031 (65.2)	1.88	1.36–2.60^***^

^a^ 8 divorced participants are categorized into the group “Bereft of spouse”

^b^ Quartile 1(Q1) is the poorest and Quartile 4(Q4) is the richest

^c^*URBMI* Urban Residents Basic Medical Insurance, *UEBMI* Urban Employees Basic Medical Insurance, *CMI* Commercial Medical Insurance

^d^40 uninsured patients are categorized into the group “others”

^e^ “Dibao” means low-income households in China, which living and medical aids from municipal governments and other welfare programs

*** *Ρ* < 0.001, ** *Ρ* < 0.01, * *Ρ* < 0.05

### Patients’ household income/expenditure, capacity to pay and OOP payments for hypertension

The mean annual household income is US$2260 (a currency exchange rate of Chinese RMB 689 Yuan to US$100 dollar, the same below), with a highest mean income of US$2427 in patients with no complication, and a lowest mean income of US$1451 in hypertensive patients with two or more complications. Mean annual household expenditure is US$2487(with a median of US$1836), and the mean household food expenditure is US$720(median value US$610). Mean OOP payment for hypertension-related healthcare is US$272 (with a median of US$53), which accounts for 15.4% of the mean household capacity to pay, and 12.1% of the mean household income. Hypertensive patients with two or more complications have highest share of household income (78.1%) and capacity to pay (53.6%), while patients with no complication have the lowest share of household income (4.3%) and capacity to pay (6.2%) (See the Table 2).

**Table 2:** Distribution of capacity to pay and OOP costs for health care across hypertensive patients in rural Shandong, China, 2016

Indicators	One complication	Two or more complications	No complication	Total
**Frequency**	1013	91	2009	3113
**Average OOP**^a^**cost of health care (US$)**^b^				
Mean	529	1133	103	272
Median	196	609	29	53
**Average annual household income**				
Mean	2000	1451	2427	2260
Median	1065	871	1306	1164
**Average annual household expenditure**				
Mean	2622	2722	2048	2487
Median	2003	2220	1741	1836
**Average annual household food expenditure**				
Mean	706	610	732	720
Median	610	610	610	610
**Average capacity to pay**^**c**^				
Mean	1916	2112	1676	1767
Median	1338	1512	1074	1161
**OOP costs share of household income(%)**	26.4	78.1	4.3	12.1
**OOP costs share of capacity to pay(%)**	27.6	53.6	6.2	15.4

*US$* United States dollar

^a^*OOP* out-of-pocket

^b^ A currency exchange rate of Chinese RMB 689 Yuan to US$100 dollar

^c^ Capacity to pay means that household expenditure minus food expenditure

### Incidence of CHE and impoverishment among patients with different numbers of complications

**Fig. 1:**
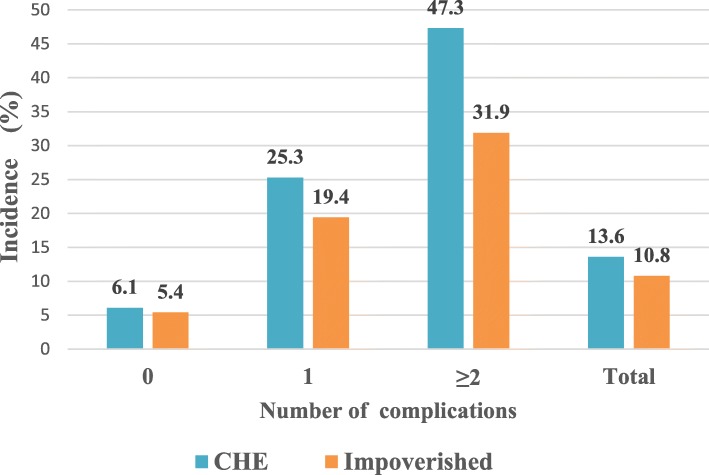
Incidence of the CHE and impoverishment for hypertensive patients in rural Shandong, China, 201

Figure 1 presents the incidence of CHE and impoverishment effect for different types of patients, of which the overall incidence of CHE and impoverishment are 13.6 and 10.8% respectively. Specifically, CHE incidence is highest among the hypertensive patients with two or more complications (47.3%), and is lowest among the patients with no complication (6.1%). Similarly, the incidence of impoverishment is highest among the hypertensive patients with two or more complications (31.9%), and is lowest among the patients with no complication (5.4%).

### CHE for hypertensive patients with different numbers of complications

In Table 3, we can observe an inverse association between CHE incidence and household income. Over 21% of patients in the poorest quartile (Q1) compared to 6.9% of those in the richest households (Q4). Similar trends are observed among all of three types of hypertensive patients. In each income level, CHE incidence is highest in the patients with two or more complications.

**Table 3:** Incidence and intensity of catastrophic health expenditure by economic status and patient composition in rural Shandong, China, 2016

CHE	No complication	One complication	Two or more complications	Total
**Head Count (HC,%)**				
Q1^a^	11.2	35.4	60.9	21.7
Q2	5.7	25.9	48.0	13.8
Q3	4.4	21.7	42.9	11.6
Q4	3.2	17.9	36.4	6.9
Total	6.1	25.3	47.3	13.6
**Mean Gap(%)**				
Q1	4.8	31.4	61.2	15.5
Q2	3.0	16.1	41.0	8.8
Q3	1.3	12.5	35.9	5.4
Q4	1.1	7.2	12.2	3.1
Total	2.5	16.8	38.0	8.3
**Mean Positive Gap (%)**				
Q1	42.9	88.5	100.5	71.4
Q2	52.0	61.9	85.4	63.8
Q3	29.5	57.7	83.7	46.6
Q4	34.4	39.8	33.5	44.9
Total	41.0	66.5	80.3	61.1

^a^ Quartile 1(Q1) is the poorest and Quartile 4(Q4) is the richest

In addition, we also use the overshoot and mean positive overshoot indicators to describe the intensity of catastrophic health expenditure. With the increase of income level, the overshoot and mean positive overshoot of CHE gradually decrease, showing a negative correlation trend. On average, health care payments for hypertension are 8.3% higher than the 40% threshold. For those experienced CHE, the MPO was found to increase to 61%.

### CHE distribution

For hypertensive patients, out-patient (13.2%) and in-patient (13.3%) are at high risk of experiencing CHE, no matter whether there is complication or not. Among the patients with no or only one kind of complication, those who are older, who with lower household income, and those with less than four family members are more likely to experience CHE. We also find that in patients with two or more complications, female and patients with lower education are inclined to suffer CHE (Table 4).

**Table 4:** Relationship between patient’s characteristics and incidence of catastrophic health expenditure in rural Shandong, China, 2016

Variable	One complication			Two or more complications			No complication		
	N (%)	χ2	***P***	N (%)	χ2	***P***	N (%)	χ2	***P***
**Age (years)**		8.66	**0.034**		4.85	0.089		25.91	**0.000**
≤ 45	10 (1.0)			0 (0.0)			50 (2.5)		
46–60	218 (21.5)			10 (11.0)			559 (27.8)		
61–70	478 (47.2)			48 (52.7)			812 (40.4)		
> 70	303 (30.3)			33 (36.3)			588 (29.3)		
**Sex**		1.10	0.294		7.97	**0.005**		1.87	0.172
Male	337 (33.3)			35 (38.5)			770 (38.3)		
Female	676 (66.7)			56 (61.5)			1239 (61.7)		
**Marital status**		15.11	**0.001**		0.00	0.991		57.73	**0.000**
Single	12 (1.2)			0 (0.0)			52 (2.6)		
Married	828 (81.7)			72 (79.1)			1671 (83.2)		
Bereft of spouse ^a^	173 (17.1)			19 (20.9)			286 (14.2)		
**Education**		1.41	0.703		9.17	**0.027**		21.13	**0.000**
None	528 (52.1)			47 (51.6)			816 (40.6)		
Primary school	314 (31.0)			25 (27.5)			718 (35.7)		
Junior school	134 (13.2)			13 (14.3)			370 (18.4)		
Senior school or above	37 (3.7)			6 (6.6)			105 (5.2)		
**Household income**		23.05	**0.000**		2.93	0.403		32.33	**0.000**
Q1^b^	279 (27.5)			35 (38.5)			469 (23.3)		
Q2	270 (26.7)			23 (25.3)			481 (23.9)		
Q3	260 (25.7)			18 (19.8)			523 (26.0)		
Q4	204 (20.1)			15 (16.5)			536 (26.7)		
**Health insurance**^**c**^		3.38	0.337		2.25	0.522		3.72	0.294
URBMI	970 (95.8)			86 (94.5)			1927 (95.9)		
UEBMI	13 (1.3)			1 (1.1)			26 (1.3)		
CMI	3 (0.3)			1 (1.1)			7 (0.3)		
Others ^d^	27 (2.7)			3 (3.1)			49 (2.4)		
**Duration of disease**		1.67	0.645		0.89	0.828		1.74	0.629
≤ 5	255 (25.2)			14 (15.4)			762 (37.9)		
6–10	319 (31.5)			32 (35.2)			699 (34.8)		
11–15	171 (16.9)			16 (17.6)			210 (10.5)		
> 15	268 (26.5)			29 (31.9)			338 (16.8)		
**In-Patient**		192.49	**0.000**		33.58	**0.000**		219.11	**0.000**
No	287 (28.3)			44 (48.4)			1929 (95.6)		
Yes	726 (71.7)			47 (51.6)			80 (4.0)		
**Out-Patient**		10.06	**0.002**		8.03	**0.005**		55.49	**0.000**
No	200 (19.7)			62 (68.1)			1829 (91.0)		
Yes	813 (80.3)			29 (31.9)			181 (9.0)		
**Dibao**^e^		18.33	**0.000**		0.01	0.932		0.04	0.839
No	68 (6.7)			78 (85.7)			1920 (95.6)		
Yes	945 (93.3)			13 (14.3)			89 (4.4)		
**Be in debt**		0.24	0.627		3.49	0.062		7.60	**0.006**
No	145 (14.3)			68 (74.7)			1771 (88.2)		
Yes	868 (85.7)			23 (25.3)			238 (11.8)		
**Head of household**		5.93	**0.015**		0.53	0.466		8.36	**0.004**
No	523 (51.6)			45 (49.5)			889 (44.3)		
Yes	490 (48.4)			46 (50.5)			1120 (55.7)		
**Head of household’s gender**		1.23	0.268		2.44	0.118		30.93	**0.000**
Male	766 (75.6)			72 (79.1)			432 (21.5)		
Female	247 (24.4)			19 (20.9)			1577 (78.5)		
**Family size**		15.23	**0.000**		3.31	0.069		0.16	0.694
≤ 4	931 (91.9)			84 (92.3)			1843 (91.7)		
> 4	82 (8.1)			7 (7.7)			166 (8.3)		
**Living arrangements**		10.93	**0.000**		1.74	0.42		79.09	**0.000**
Empty-nest single	156 (15.4)			15 (16.5)			269 (13.4)		
Empty-nest couple	670 (66.1)			63 (69.2)			1298 (64.6)		
Others	187 (18.5)			13 (14.3)			442 (22.0)		

The *P*-values indicate statistical significance at 5% level

^a^ 8 divorced participants are categorized into the group “Bereft of spouse”

^b^ Quartile 1(Q1) is the poorest and Quartile 4(Q4) is the richest

^c^*URBMI* Urban Residents Basic Medical Insurance, *UEBMI* Urban Employees Basic Medical Insurance, *CMI* Commercial Medical Insurance

^d^ 40 uninsured patients are categorized into the group “others”;

^e^ “Dibao” means low-income households in China, which living and medical aids from municipal governments and other welfare programs

### Determinants of CHE in patients with different numbers of complications

For better understanding of the determinants of CHE in each subgroup, we use a logistic regression model to examine the related factors of CHE for each of the three subgroups separately in Table 5. Two factors are found to be statistically significant (*P* < 0.05) in all three types of patients, including outpatient service use in the past 6 months, and inpatient service use in the 12 months. Among the patients with no complication, those empty-nest singles and with lower education level are more likely to experience CHE (P < 0.05). Likewise, those who have lower household income are more likely to experience CHE in patients with one complication (P < 0.05).

**Table 5:** Logistic regression model of determinants of CHE for health care of different kinds of complications among hypertensive patients in rural Shandong, China, 2016

Variables	One complication			Two or more complications			No complication		
	OR	95%CI	***P***	OR	95%CI	***P***	OR	95%CI	***P***
**Household income**						NA ^a^			NA
Q4	1.0								
Q1^b^	2.18	1.25–3.78	**0.006**						
Q2	1.39	0.81–2.41	0.228						
Q3	1.06	0.62–1.81	0.839						
**In-Patient**									
No	1.0			1.0			1.0		
Yes	9.14	6.49–12.89	**0.000**	40.96	7.72–217.30	**0.000**	20.10	11.47–35.33	**0.000**
**Out-Patient**									
No	1.0			1.0			1.0		
Yes	1.69	1.13–2.53	**0.011**	8.44	1.16–61.43	**0.035**	5.62	3.39–9.32	**0.000**
**Dibao**^**c**^						NA			NA
No	1.0								
Yes	2.52	1.39–4.55	**0.002**						
**Education**			NA			NA			
None							1.0		
Primary school							0.50	0.31–0.82	**0.005**
Junior school							0.39	0.18–0.86	**0.019**
Senior school or above							0.69	0.24–1.98	0.482
**Living arrangements**			NA			NA			
Others							1.0		
Empty-nest single							5.88	1.48–23.39	**0.012**
Empty-nest couple							1.82	0.61–5.38	0.284
Observations	1013			91		2009			
Adjusted R^2^	0.32			0.61		0.28			

The *P*- values indicate statistical significance at 5% level

^a^ not applicable

^b^ Quartile 1(Q1) is the poorest and Quartile 4(Q4) is the richest

^c^ “Dibao” means low-income households in China, which living and medical aids from municipal governments and other welfare programs

## Discussion

The participants in this study were selected from the chronic patients’ management system in the sampling villages, which were randomly chosen from those selected counties based on GDP per capita. This sampling process enables its representativeness of the rural Shandong, China. This survey based on villages offers an insight into the economic burden shouldered by the hypertensive patients and their households in rural Shandong, China.

In this study, the incidence of CHE is 13.6%. In order to better understand the incidence of CHE, we try to compare the CHE in this study to some others in China which used the same measuring method of CHE as ours. It is higher than the reported 6.7, 11.19 and 5.4% in general households in rural Sichuan, rural Yunnan and rural Shaanxi respectively [36–38] . A study by Zhao et al. estimated an incidence of 6.3 and 5.4% in general households in rural Gansu and Heilongjiang, which were both lower than the incidence in this study [39]. Another study conducted in the same province as this study indicated that 4.5 and 4.3% of the rural general households experienced CHE in 2006 and 2008, which were also lower than the incidence of CHE in this study [40] . Similarly, the incidence of impoverishment in the current study is 10.8%, which is higher than the 7.0% found in a study using the National Health Survey data in 2008 and 2.6% in another study using the 2000 Chinese National Bureau of Statistics data [23, 41]. Even though the incidence of CHE in this study is lower the reported 23.48% in hypertensive patients’ households in rural Shaanxi in 2013 [42], the results both from Shandong and Shaanxi indicate that hypertension might exert a significant effect to incur CHE and poverty on the patients and their households in rural China. In addition, the Chinese government has taken several measures to promote essential public health service (including hypertension case management) and also universal health coverage in the past 5 years. The gap in CHE among hypertensive patients between Shandong and Shaanxi province, might imply a progress in such measures.

Not surprisingly, the results of this study reveal that the hypertensive patients with complication are more likely to experience CHE compared with those without complication. In addition, with the increase in the types of hypertensive complications, the intensity and incidence of CHE also increase significantly. Some previous studies indicate that the treatment and healthcare costs for hypertensive complications (i.e., coronary heart disease, stroke) are particularly expensive [40] .The high economic burden for hypertension patients and their households mainly comes from hypertensive complications [43] . This finding implies a need for the community health providers to monitor the blood pressure regularly for the rural hypertensive patients, so as to prevent the progression of the hypertension and the occurrence of its complications. The primary goal of health insurance scheme is to prevent patients from falling into poverty trap due to healthcare expenditures worldwide. However, in this study, we found that health insurance has no significant effect on the prevention of CHE, which is similar with some previous studies [44–47]. This finding indicates that the healthcare expenditure for hypertension after reimbursement still poses a high financial burden on the patients’ households. In other words, the protection effect of the health insurance scheme on the hypertension and its complications is very limited. Therefore, to upgrade the height of coverage of the health insurance schemes targeting the hypertensive patients, especially for those patients with complications, is essential to decrease the possibility of CHE in the hypertensive patients’ households. A previous study on Global Budget Payment System (GBPS) has shown that it can help reduce total medical expense, and OOP expenditure significantly. Therefore, to explore new targeting medical insurance payment system is also important to reduce the incidence of CHE on hypertension and its complications [48].

Similarly, our results find that the outpatient and hospitalization service use are the determinants of CHE in hypertensive patients [23, 49] . A study in Yichang, China found that average expense for each hypertension-related outpatient and inpatient visit was US$ 36.1 and US$ 1877.0 respectively [50] . Hypertensive patients need to use outpatient visit for about eight times per year [51] . The health service utilization posed a high direct economic burden on the patients and their households. This was also demonstrated to be true in some other countries [52, 53]. In addition, some other indirect costs, such as transportation and accommodation expenses for health service use, and formal care expenses for those need care, also bring dramatic burden to the patients and their households. This finding suggests for the policy makers to develop interventions of healthcare cost control, especially for those inpatient services, to reduce healthcare expenditure for the hypertensive patients and their households. For example, to improve the two-way referral system and its supporting measures would probably reduce total healthcare expenditure in inpatient and outpatient use among the hypertensive patients [54]. Simultaneously, practitioners in community health centers should carry out hypertensive patients targeting health education, especially in patients with poor education, to improve their understanding of hypertension and its consequences so as to prevent the progression of the hypertension and its complications.

Consistent with the studies in some other diseases, this study also reveals that the incidence and intensity of CHE among hypertensive patients is negatively correlated with the economic status, patients in lower income quartiles are at higher risk of experiencing CHE. In the same income quartile, the incidence and intensity of CHE increased with the increase in the number of hypertension complications. China began to implement a strategy of Targeted-Poverty-Alleviation in 2014, and the disease-caused poverty is the most common target in this strategy. This finding is meaningful to develop anti-poverty policy of pro-patients with chronic diseases (e.g., hypertension), especially for those with complications, so as to effectively achieve the goal of the Targeted-Poverty-Alleviation strategy.

Several limitations are involved in this study. First, the number of patients with two or more complications is only 91(3.0%). Of the participants, 63.3% were women. These might result in possible bias. Second, when we collect the expenditure and income of the patient’s household (such as OOP, food expenditure), even though we have tried to, it is hard to exclude recall bias.

## Conclusion

This study reveals that the incidence and intensity of CHE are relatively high in hypertensive patients in rural China. Patients with complications are at higher risk of experiencing CHE than those with no complications. Our findings also show that patients who use outpatient or inpatient services are more likely to suffer from CHE. In addition, some other at-risk factors, including living arrangements, family size and education level, are also identified in some subgroups. The results imply a need of regular monitoring for blood pressure in rural hypertensive patients. Some measures to enhance adherence to anti-hypertension treatment as well as lifestyle changes to lower BP (blood pressure), are also of significance to prevent the progression of the hypertension and its complications, so as to reduce the risk of potential CHE.

## Data Availability

The datasets used and/or analyzed during the current study are available from the corresponding author on reasonable request.

## Abbreviations

CHE: Catastrophic health expenditure
WHO: World health organization
OOP: Out-of-pocket
GDP: Gross Domestic Product
SBP: Systolic blood pressure
DBP: Diastolic blood pressure
